# Transplantation of endothelial progenitor cells overexpressing mir‐126‐3p improves vascular repair in a diabetic rat model

**DOI:** 10.1002/mco2.224

**Published:** 2023-03-03

**Authors:** Guangchen He, Haitao Lu, Yueqi Zhu, Yuehua Li, Liming Wei

**Affiliations:** ^1^ Department of Radiology Shanghai Sixth People's Hospital Affiliated to Shanghai Jiao Tong University School of Medicine Shanghai China

Dear Editor,

Endovascular recanalization was considered as a first‐line treatment for diabetic patients with severe peripheral artery stenosis. The current treatment strategies, which include employing drug‐eluting balloons and drug‐eluting stents, have been shown to successfully achieve revascularization and relieve symptoms. However, long‐term patency is often a challenge due to high rate of early restenosis (up to 33%), especially in infrapopliteal arteries. The pathogenesis might be attributed to inflammatory activation and endothelial dysfunction leading to neointimal hyperplasia and restenosis.

Endothelial progenitor cell (EPC) transplantation was as a promising strategy for endothelial repair in peripheral artery diseases. EPCs are bone marrow‐derived late‐stage stem cells found circulating in the blood and possess the ability to proliferate and differentiate into endothelial cells, thereby contributing to vascular repair and angiogenesis.^1^ Recently, accumulating evidences suggested that microRNAs (miRNAs) could regulate the proliferation, migration, and angiogenesis of EPCs. miR‐126‐3p modulates the expression of proangiogenic cytokines, thereby regulating angiogenesis and the homing and retention of EPCs in injured vascular walls.^2^ Deficiency of miR‐126 in diabetic EPCs was also associated with EPC functional disorders. Therefore, we investigated whether miR‐126‐3p overexpression could restore EPC biology and improve vascular repair in type 1 diabetes mellitus (T1DM).

EPCs originated from the bone marrow of T1DM rats and healthy controls were isolated by density gradient centrifugation and grown in endothelial growth medium 2 (EGM‐2) media. The attached EPCs elongated and became spindle‐shaped after media changes on the 4th and 7th days of culture. Cellular immunostaining studies showed the binding of adherent cells to uptake FITC‐Ulex Europaeus Agglutinin I (FITC‐UEA‐I) and Dil‐acetylated low‐density lipoprotein (Dil‐ac‐LDL) (Figure 1A). Meanwhile, early EPCs expressed endothelial markers (CD34 and CD133) at a comparable level (Figure S1). EPCs were transfected with the recombinant lentiviral expression vector using reverse transcription quantitative polymerase chain reaction (RT‐qPCR) analysis to confirm a significant relative upregulation of miR‐126‐3p (Figure S2).

**FIGURE 1:**
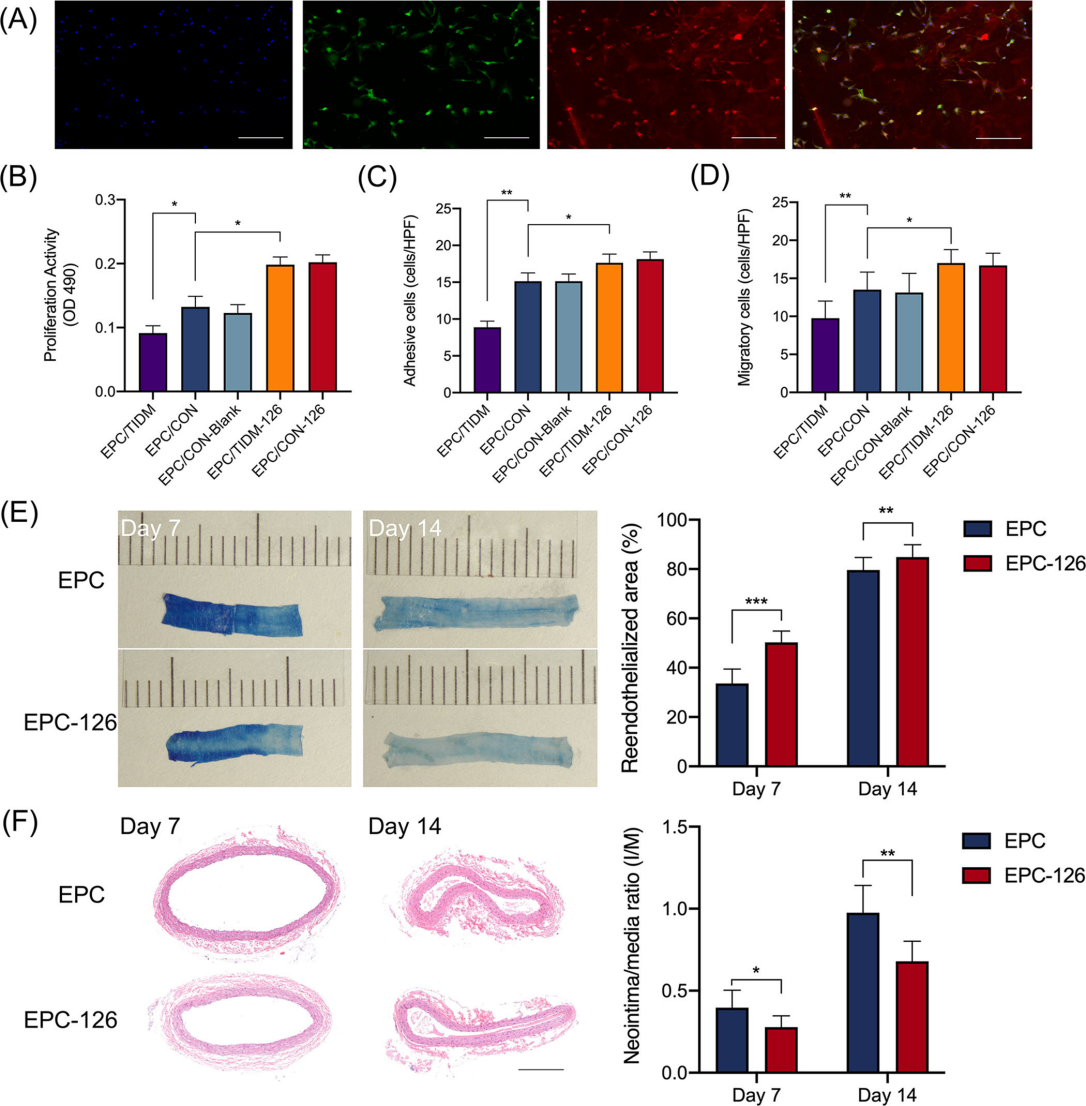
Characterization, function, and effect of endothelial progenitor cells (EPCs) on reendothelialization. (A) Characterization of EPCs derived from rat bone marrow. Nuclei were counterstained with 0.5 µg/mL DAPI (blue); cells were stained with FITC‐Ulex Europaeus Agglutinin I (FITC‐UEA‐I) (green) and Dil‐acetylated low‐density lipoprotein (Dil‐ac‐LDL) (red) and merged. The proliferation (B), adhesion (C), and migration (D) of EPCs after miR‐126‐3p overexpression in vitro. (E) Evans blue dye staining of whole‐mount enface carotid arteries at 7 and 14 days after carotid balloon surgery. Non‐endothelialized lesions were marked by blue staining and the reendothelialized area appeared white. Reendothelialization was quantified as white‐area/total‐area ratio. (F) Sections of carotid arteries at 7 and 14 days after injury with hematoxylin and eosin staining measured with intima‐area/media‐area ratio (I/M). Data are given as mean ± SD. ^*^ *p* < 0.05, ^**^ *p* < 0.01, ^***^ *p* < 0.001. HPF, high power field. Scale bar: 400 µm.

Successfully isolated and transfected EPCs were allocated into an EPC/T1DM group (EPC from T1DM rats), an EPC/CON group (EPC from healthy controls), an EPC/CON‐Blank group (EPC from healthy controls transfected with a blank vector), an EPC/T1DM‐126 group, or an EPC/CON‐126 group (EPCs from T1DM rats or healthy controls transfected with miR‐126‐3p). The proliferation of EPCs was assessed by MTT (3‐[4,5‐dimethylthiazol‐2‐yl]‐2,5 diphenyl tetrazolium bromide) assays. There was a significant decrease in cell proliferation in the EPCs isolated from T1DM rats compared with healthy controls (*p* = 0.023, Figure 1B). Nevertheless, the proliferation improved after transfection with miR‐126‐3p (*p* = 0.012). Similarly, the adhesive activity of T1DM EPCs showed a significant decrease (8.2 ± 1.5) when compared with EPCs from healthy controls (14.9 ± 1.4, *p* = 0.003, Figure 1C). The decrease was reversed in EPC‐126 (17.8 ± 1.8), which showed a significant increase in adhesive activity compared with healthy controls (14.9 ± 1.4, *p* = 0.021). The migratory capacity of EPCs was evaluated using modified Boyden chamber assays. The results indicated that EPC migration was significantly impaired in T1DM rats (9.8 ± 2.3) compared with healthy controls (13.5 ± 2.3, *p* = 0.002, Figure 1D). miR‐126‐3p significantly increased EPC migration (17 ± 1.8) when compared with controls (13.5 ± 2.3, *p* = 0.001). These results showed that the proliferation, adhesion, and migration capacities of EPCs were significantly decreased in T1DM rats and were enhanced after transfection with miR‐126.

At 7 and 14 days after injury, reendothelialization was evaluated by staining of the Evans blue dye and was presented as percentage of reendothelialization area (defined as area not stained with Evans blue/total injured surface area). As expected, reendothelialization was significantly greater in the EPC‐126‐infected group than in the EPC‐treated group (50.3 ± 4.7% vs. 33.6 ± 5.8% at day 7, *p* < 0.001; 84.9 ± 5.1% vs. 79.6 ± 5.7% at day 14, *p* = 0.003, Figure 1E). For intimal hyperplasia analysis, sections were stained with hematoxylin and eosin. A marked reduction in intima/media ratio was found in the EPC‐126‐treated group (0.278 ± 0.071 at day 7 and 0.683 ± 0.122 at day 14), whereas the intima/media ratios in the EPC group were 0.396 ± 0.107 at day 7 and 0.975 ± 0.167 at day 14 (*p* < 0.05 vs. EPC‐126, Figure 1F). These results suggested that modulation of EPCs with overexpression of miR‐126 could reduce neointimal hyperplasia and accelerate reendothelialization.

EPCs are precursor cells derived from bone marrow and can proliferate and differentiate into vascular endothelial cells. There is growing evidence that EPCs are involved in the maintenance of normal endothelial function and angiogenesis. Previous studies have shown that the transfusion of exogenous EPCs can contribute to improved reendothelialization, neovascularization, and endothelial repair following balloon injury. However, reduced circulating EPC numbers and EPC dysfunction were found in diabetic patients. The EPC recruitment at reendothelialization sites following arterial injury was also impaired in T1DM, likely contributing to diabetic vasculopathy. Initially, we found that the EPCs in T1DM rats showed a significant decrease in number and impaired function when compared with normal controls in in vitro cell function assays. The EPCs mobilized into peripheral circulation participated in the process of vascular repair, while the poor functional state of EPC obliterates its potentiality to repair carotid injury.

Furthermore, miR‐126 was abundantly expressed in endothelial cells and crucial for maintaining vascular homeostasis. EPCs derived from DM patients reportedly had lower expression of miR‐126, leading to their functional impairment. Overexpression of miR‐126 increased migration and promoted homing and stemness maintenance in EPCs. Hence, modulation of EPC function is a potential therapeutic target in exogenous EPC transplantation. To prove it, we successfully transfected EPCs with miR‐126 using lentiviral expression vectors and the expression status was confirmed by RT‐qPCR analysis. The miR‐126‐overexpressing EPCs showed improved proliferative, adhesive, and migratory functions. Because miR‐126 is specifically expressed in endothelial cells, the overexpression of miR‐126 could positively activate the vascular endothelial growth factor (VEGF)‐dependent signaling pathways and directly affect Spred‐1, Syndecan‐1, vascular cell adhesion molecule‐1 (VCAM1), and phosphoinositide‐3‐kinase regulatory subunit 2 (PIK3R2).^3^, ^4^ Inhibiting the expression of Spred‐1 and PIK3R2 would enhance VEGF and fibroblast growth factor (FGF)‐induced angiogenesis.

We further produced a carotid artery injury model in T1DM rats, which were transplanted with EPCs overexpressing miR‐126‐3p, and evaluated for improvement in reendothelialization. As expected, the EPC‐126 was incorporated into injured intima and improved reendothelialization after carotid artery injury. The reestablishment of an intact endothelium after injury is an important step in the prevention of neointimal hyperplasia and stent thrombosis. A previous study postulated that miR‐126 promotes EPC function by modulating the expression of stromal cell‐derived factor 1 (SDF‐1) via repression of the regulator of G protein signaling 16 (RGS16).^5^ RGS16 silencing enabled chemokine receptor 4 (CXCR4) to mediate autoregulation and increase the expression of SDF‐1. Pei et al.^2^ showed that miR‐126 could also directly increased the production of CXCR4 and enhanced the function of EPCs in diabetic rats. Hence, miR‐126 functioned in a complexed regulatory signaling network of EPCs that subsequently facilitated reendothelialization in endothelial injury.

In conclusion, our study demonstrates that miR‐126‐3p could restore the biology of EPCs from diabetic rats. The transplantation of miR‐126‐3p‐overexpressing EPCs promoted reendothelialization in carotid artery injury models by upregulation of the endothelial cell function.

## AUTHOR CONTRIBUTIONS

G.H. and L.W. conceived and drafted the manuscript. G.H. drew the figures. L.W. and Y.L. discussed the concepts of the manuscript. H.L. and Y.Z. provided valuable discussion and revised the manuscript. All authors contributed to the article and approved the submitted version.

## CONFLICT OF INTEREST STATMENT

All the authors declare no conflicts of interest.

## ETHICS STATEMENT

This project was permitted and approved by the Committee of the Sixth People's Hospital at the Shanghai Jiao Tong University (License: SCXK2018‐0006).

## Supporting information

Supporting Information

## Data Availability

All data are available from the corresponding author upon request.
