# Supplementary material for: Transplantation of endothelial progenitor cells overexpressing mir‐126‐3p improves vascular repair in a diabetic rat model
Source: MedComm (2020). 2023 Mar 3;4(2):e224. doi: 10.1002/mco2.224 (PMC9983319; doi:10.1002/mco2.224)

**Supplementary Materials**

**Transplantation of Endothelial Progenitor Cells Overexpressing miR-126-3p Improves Vascular Repair in a Diabetic Rat Model**

Guangchen He^1^ M.D., Haitao Lu^1^ M.D., Yueqi Zhu^1^ M.D., Yuehua Li*^1^ M.D., Liming Wei*^1^ M.D.

^1^Department of Radiology, Shanghai Sixth People's Hospital Affiliated to Shanghai Jiao Tong University School of Medicine; No. 600, Yi Shan Road; Shanghai, 200233, China

**Corresponding Author**: Liming Wei M.D. and Yuehua Li M.D.

Address: No. 600, Yishan Road; Shanghai, 200233, China

E-Mail: weilimingnj@126.com and liyuehua312@163.com

Tel.: +86-21-66301136; Fax: +86-21-66303983

**Materials and Methods**

*EPC culture and characterization*

EPCs from bone marrow mononuclear cells (BMMCs) of healthy controls (CON) and T1DM rats were isolated and cultured in accordance with previously mentioned methods.^1^ In brief, Sprague-Dawley (SD) rats (male, 150-200 g, SPF) supplied by Shanghai Xipuer-Bikai Experimental Animal Co., Ltd (Shanghai, China) were decapitated under anesthesia and soaked in 75% ethanol for 15 min. After removing femur and tibia of both sides, the bone marrow cavity was rinsed repeatedly with 10 mL of PBS (4°C, pH 7.3). The samples were centrifuged and isolated mononuclear cells (MNCs) using Histopaque-1083 (Sigma-Aldrich, St. Louis, MO, USA). Isolated MNCs was resuspended in endothelial cell basal medium-2 (EGM-2) BulletKit system (Clonetics, Walkersville, MD, USA) and seeded on plates coated with fibronectin (Sigma, St. Louis, MO, USA). After 3 days, non-adherent cells were removed. The cultures were maintained through day 7, and adherent cells (considered as EPCs) were harvested for further studies. The characteristic colony-forming units (CFU) of endothelial cells (ECs) were observed on day 7 of EPC culture. EPCs were stained with acetylated low-density lipoprotein (Dil-Ac-LDL; Invitrogen, Carlsbad, CA, USA) incubated with fluorescein isothiocyanate (FITC)-conjugated *Ulex europaeus* agglutinin-1 (UEA-1; Sigma, St Louis, MO, USA). Cells double-positive for both Dil-Ac-LDL and FITC-conjugated lectin were identified as differentiating EPCs. Double-positive cells were counted using a phase contrast fluorescence microscope (Olympus, USA). After resuspension in fluorescence-activated cell sorting buffer (500 μL), cells were analyzed by flow cytometry (BD LSRFortassa; Becton Dickinson, NJ, USA) with BD FACSDiva software (version 8.0).

*RNA isolation and miRNA real-time PCR*

Trizol reagent (Invitrogen) was used to extracted total RNA from cells. A ccording to the manufacturer’s instructions, the extracted RNA was further purified and reverse-transcribed using the TaqMan MiRNA reverse transcription kit (Applied Biosystems) using primers 5′-ACAACTTTGGTATCGTGGAAGG-3′ and 5′-GCCATCACGCCACAGTTTC-3′.

The conditions for the PCR were: stage 1, 95°C for 10 min and stage 2, 95°C for 15 s and 60°C for 60 s. Stage 2 was repeated for 40 cycles. Real-time PCR was performed using SYBR Green PCR master mix with the Applied Biosystems 7900HT fast real-time PCR System. *U6* was used as the endogenous control. All samples were normalized to internal controls, and the relative expression level was calculated using the 2^−ΔΔCt^ analysis method.

*Transfection of miR-126*

EPCs from healthy controls were allocated into a control group (CON), a blank vector group (Blank), or a miR-126 transfection group (miR-126). Considering only the 3p brand is active for revascularization in endothelial cells that only the miR-126-3p,^2^ the lentiviral expression vectors, mCherry-miR-126-3p (Clontech Laboratories, CA, USA) and mCherry (Invitrogen, Carlsbad, CA, USA), were constructed to stably overexpress the mature sequences of miR-126-3p and mCherry in EPCs. The mature miR-126-3p sequence was subcloned into *Eco*R1 and *Bam*H1 sites of the pLVX-IRES-mCherry vector. EPCs were transfected with the lentiviral expression vectors at a multiplicity of infection (MOI) value of 100 in 6-well culture plates, at a density of 5×10^4^ cells per well. The miR-126-3p expression was analyzed using quantitative real-time PCR.

*Functional assays of EPCs*

The EPC function assay was performed as previously described.^3^ The EPCs of the control, blank and miR-126-3p transfection groups were digested with 0.25% trypsin and cultured in medium of serum-free in a 96-well culture plate (200 μL/well). After 24 h, 10 μL of 3-(4,5-dimethylthiazol-2-yl)-2,5-diphenyltetrazolium bromide (MTT, 5 g/L; Fluka) was added and the EPCs were incubated for another 4 h. After aspirating the supernatant was discarded, 200 μL dimethyl sulfoxide was added to the EPCs and shaken for 10 min. The optical density was measured at 490 nm. EPCs (2.5×10^4^ /well) were seeded in 6-well plates and incubated for 30 min. After three washes with PBS, the attached cells were counted. The adhesion assay was condcted by evaluating the mean number of attached cells per well in five high-power fields (200×). The migration assay was peformed in a modified Boyden chamber. After 7 days of culture, 3×10^4^ EPCs were placed in the upper well of the modified Boyden chamber (Corning Costar) for 12 h. The lower well of the chamber contained EBM-2 and recombinant VEGF165 (50 ng/mL; Sigma). Hoechst 33258 (Sigma, USA) was used to stain the nuclei of cells that migrated into the lower chamber. Cells were counted manually in five random microscopic fields (200×) from each well. The process was repeated 8 times for each experiment of the functional assays.

*Transplantation of EPCs for balloon-induced vascular injury*

All procedures were approved by the Animal Research Committee of the Sixth People’s Hospital at the Shanghai Jiao Tong University. Male SD rats (250–300 g) were injected intraperitoneally with a 50 mg/kg single dose of STZ (Sigma-Aldrich, St. Louis, MO, USA) for induction of T1DM. After 4 weeks, only animals with fasting blood glucose levels >16.7 mmol/L of blood glucose were classified as diabetic. To construct a vascular injury model, the DM rats were anesthetized with 75 mg/kg pentobarbital. The left external carotid was separated and introduced a 2F Fogarty arterial embolectomy balloon catheter (Edwards Lifesciences, Irvine, CA, USA) with 100 U/kg heparin sodium. The balloon was distended with saline and pulled back three times to induce endothelial injury in the common carotid artery. After injury, 3×10^6^ EPCs were injected intravenously. On days 7 and 14, the rats were anesthetized and intravenously injected evans blue dye (5%). After 10 min, the rats were euthanized to harvest left common carotid artery. Non-endothelialized lesions were marked by blue staining, whereas the re-endothelialized area appeared white. All arteries were taken starting from bifurcation with a length of 1 cm. Re-endothelialization was quantified as white-area/total-area ratio.

*Histological analysis*

Histological analysis was performed as previously described.^4^ The left carotid arteries were cut systematically in sections of 5-μm thickness and stained with hematoxylin and eosin. Areas of intima, media, and the intima/media ratio were measured. Images were obtained and analyzed in a microscope (Leica CM3050S, Leica Microsystems, Germany) and LEICA Application Suite software (version 3.8).

*Statistical analysis*

All data are reported as the mean ± SD. Statistical significance was evaluated by Student’s *t*-test or a one-way ANOVA followed by the Tukey–Kramer *post-hoc* test to compare between more than two groups. All statistical analyses were performed using SPSS statistical software (SPSS 23; SPSS Inc., Chicago, IL, USA).

**Reference**

1. Hill JM, Zalos G, Halcox JP, Schenke WH, Waclawiw MA, Quyyumi AA, et al. Circulating endothelial progenitor cells, vascular function, and cardiovascular risk. *N Engl J Med*. 2003;348:593-600

2. Bassand K, Metzinger L, Naïm M, Mouhoubi N, Haddad O, Assoun V, et al. Mir-126-3p is essential for cxcl12-induced angiogenesis. *J Cell Mol Med*. 2021;25:6032-6045

3. Xu MG, Wang JM, Chen L, Wang Y, Yang Z, Tao J. Berberine-induced upregulation of circulating endothelial progenitor cells is related to nitric oxide production in healthy subjects. *Cardiology*. 2009;112:279-286

4. Li X, Chen C, Wei L, Li Q, Niu X, Xu Y, et al. Exosomes derived from endothelial progenitor cells attenuate vascular repair and accelerate reendothelialization by enhancing endothelial function. *Cytotherapy*. 2016;18:253-262

**Figure legends**

Figure S1. Characterization of EPCs derived from rat bone marrow. (A) Flow chart of study design. (B) The morphology of EPCs on day 7 of culture. (C) Markers of EPCs detected by flow cytometry analysis using CD34 and CD133. (D) Percentage of cells combined FITC-UEA-l and Dil-ac-LDL labeling. Scale bar: 100 μm.

Figure S2. The transfection efficiency and miR-126-3p overexpression in EPCs. (A) The background of EPCs transfected with mCherry-miR-126-3p. (B) EPCs expressing mCherry (red). (C) Quantitative analysis of transfection efficiency of lentiviral expression vectors. (D) The transfection efficiency of miR-126 was confirmed by real-time PCR. Values represent means ± SD. * *P* < 0.05, ** *P* < 0.01, *** *P* < 0.001. Scale bar: 200 μm.


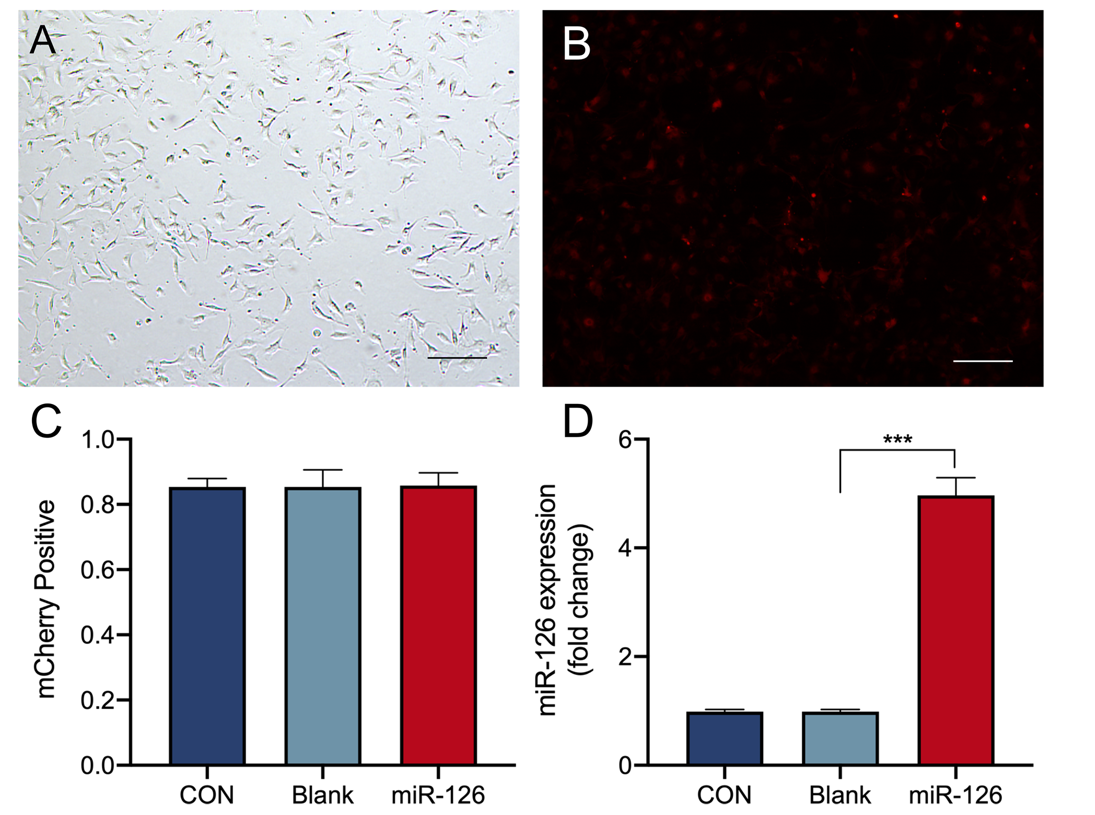

Supplement: Supplementary file 1 — Supporting Information [file MCO2-4-e224-s001.docx]
